# Multiple Myeloma with Hypoechoic Liver Nodules

**DOI:** 10.5334/jbsr.1856

**Published:** 2019-10-02

**Authors:** Mathilde Duesberg, Pierre Bosschaert

**Affiliations:** 1 Clinique St-Pierre, BE

**Keywords:** multiple myeloma, extramedullary, liver, nodular lesions

## Abstract

**Teaching point:** In the context of malignancy with lytic bone lesions, strongly hypoechoic liver nodules should alert for the possible diagnosis of extramedullary multiple myeloma.

## Case Report

An 81-year-old man was found to have multiple bone lesions on a thoracic computed tomography (CT) in the context of pneumonia. The patient only reported a mild weight loss and ordinary low back pain. His physical examination was unremarkable. Routine laboratory tests results were normal.

**Figure 1:**
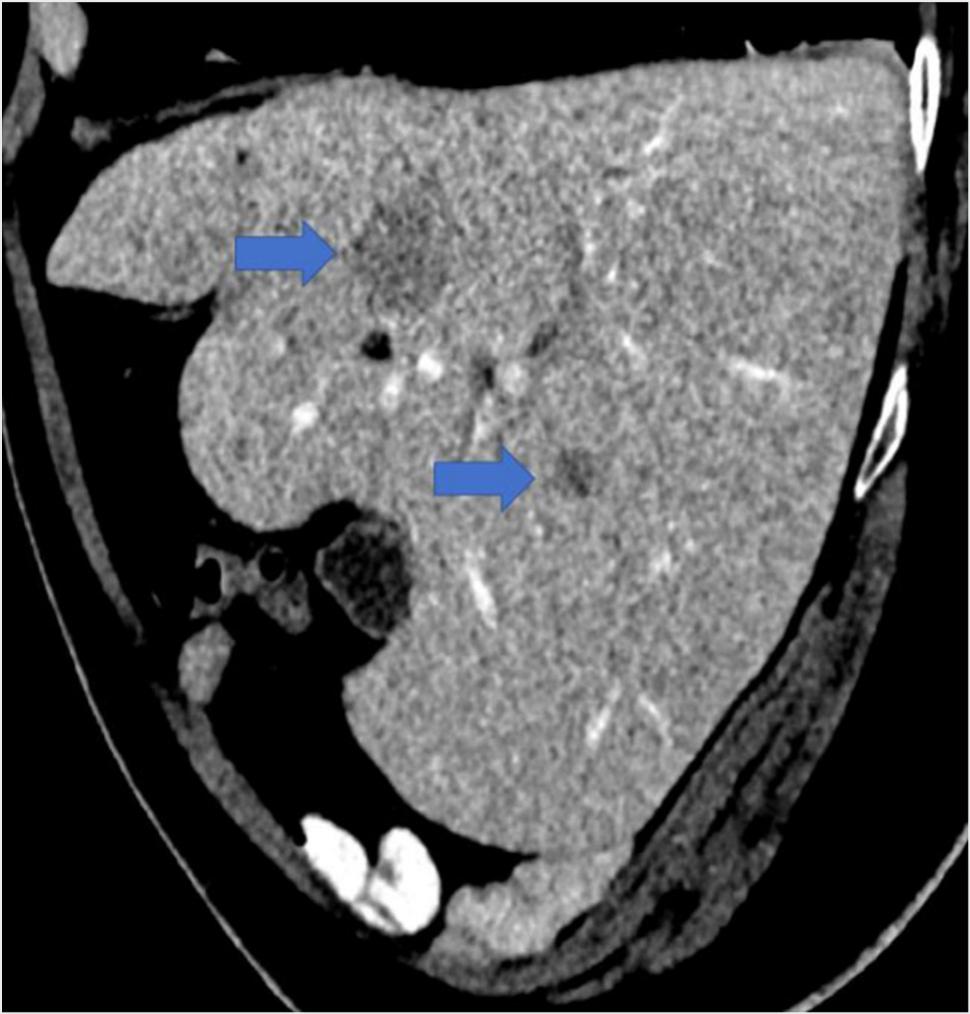


Abdominal contrast-enhanced computed tomography (CECT) showed disseminated osteolytic bone lesions and two barely visible hypoattenuating liver nodules (arrows, Figure 1).

**Figure 2:**
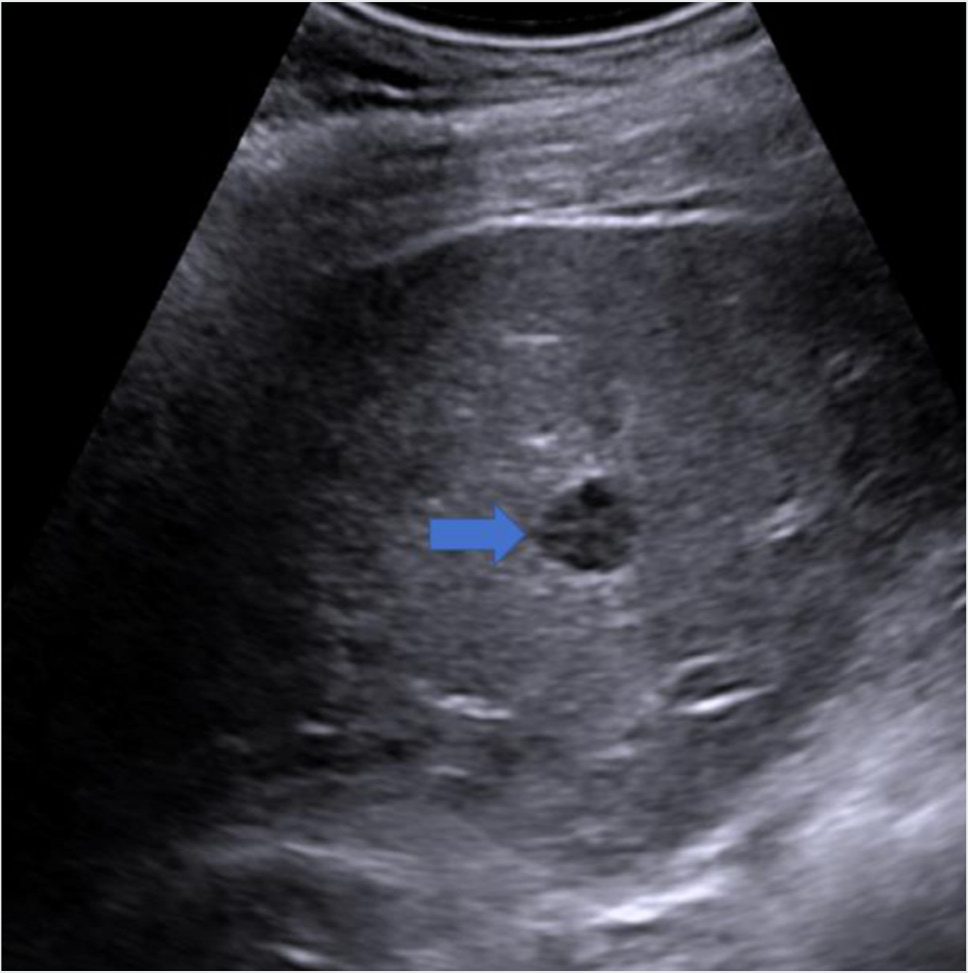


Ultrasound (US) of the hepato-biliary tract confirmed two homogeneous strongly hypoechoic liver nodules, measuring 20 and 12 mm in diameter. The lesions’ margins were well-defined, with neither hypoechoic halo sign, nor posterior acoustic enhancement or shadowing (arrow, Figure 2).

**Figure 3:**
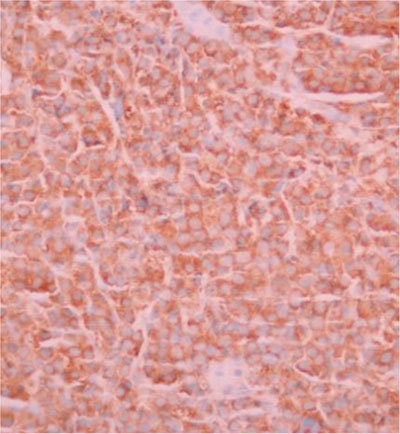


Since no primary tumor was identified, percutaneous US-guided fine-needle biopsy of one of the liver lesions was performed, which revealed unexpectedly extramedullary location of multiple myeloma (MM). The immunochemistry staining of kappa light chain was positive (Figure 3).

**Figure 4:**
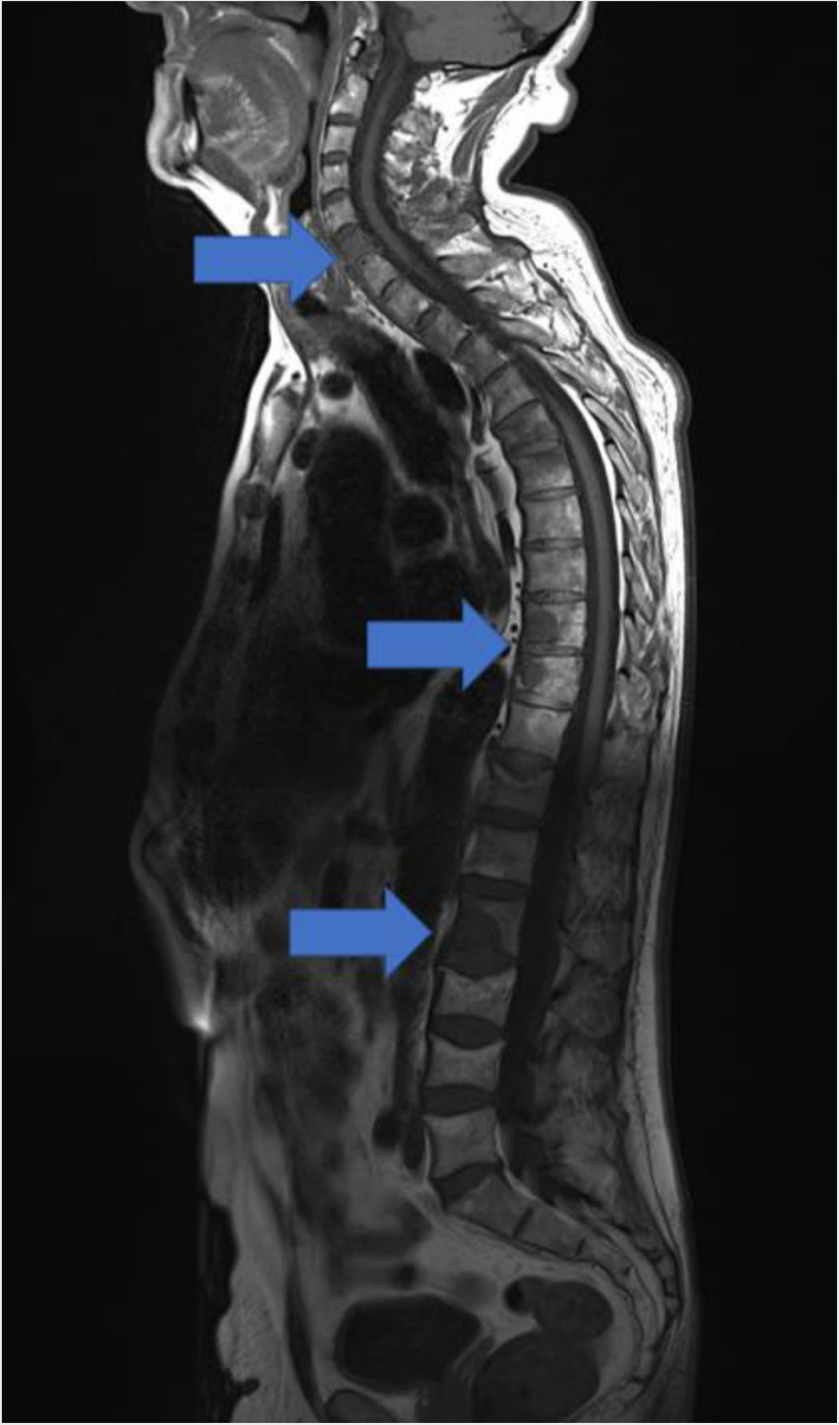


To illustrate the imaging characteristics of the bone lesions, a magnetic resonance imaging (MRI) was realized and confirmed malignant marrow-replacing lesions, especially on the spine (arrows, Figure 4).

## Comment

Multiple myeloma (MM) is the most common primary bone malignancy among adults. Soft tissue involvement of MM is uncommon upon diagnosis and is referred to as extramedullary myeloma (EM) [1]. There are two histological patterns of hepatic myeloma:

- Diffuse plasma cell infiltration of the liver, usually reported as autopsy findings in 40% of patients dying with MM and radiologically silent.
- Nodular liver presentation is a rare condition with an incidence of 0.35%. Only 27 cases have been reported so far in the scientific literature. The lesions are often described as very hypoechoic on US and hypoattenuating on CT. This was the case for our patient. This presentation usually indicates advanced stage of the disease with aggressive clinical course and poor prognosis.

The approach to a patient with strongly hypoechoic liver nodules remains challenging. The differential diagnosis is broad and includes: benign focal liver lesions, hepatocellular or cholangiocellular carcinomas, metastatic carcinomas, metastatic endocrine tumors, and lymphoma. Although uncommon, EM should be included in the differential diagnosis, especially in the context of associated lytic bone lesions.
